# Air–liquid interface enhances oxidative phosphorylation in intestinal epithelial cell line IPEC-J2

**DOI:** 10.1038/cddiscovery.2017.1

**Published:** 2017-02-27

**Authors:** Sonja Klasvogt, Werner Zuschratter, Anke Schmidt, Andrea Kröber, Sandra Vorwerk, Romina Wolter, Berend Isermann, Klaus Wimmers, Hermann-Josef Rothkötter, Constanze Nossol

**Affiliations:** 1 Institute of Anatomy, Otto-von-Guericke University, Magdeburg 39120, Germany; 2 Leibniz Institute for Neurobiology, Otto-von-Guericke University, Magdeburg 39120, Germany; 3 Institute of Clinical Chemistry and Pathochemistry, Otto-von-Guericke University, Magdeburg 39120, Germany; 4 Leibniz Institute for Farm Animal Biology, Dummerstorf 18196, Germany

## Abstract

The intestinal porcine epithelial cell line IPEC-J2, cultured under the air–liquid interface (ALI) conditions, develops remarkable morphological characteristics close to intestinal epithelial cells *in vivo*. Improved oxygen availability has been hypothesised to be the leading cause of this morphological differentiation. We assessed oxygen availability in ALI cultures and examined the influence of this cell culture method on glycolysis and oxidative phosphorylation in IPEC-J2 using the submerged membrane culture (SMC) and ALI cultures. Furthermore, the role of HIF-1 as mediator of oxygen availability was analysed. Measurements of oxygen tension confirmed increased oxygen availability at the medium–cell interface and demonstrated reduced oxygen extraction at the basal compartment in ALI. Microarray analysis to determine changes in the genetic profile of IPEC-J2 in ALI identified 2751 modified transcripts. Further examinations of candidate genes revealed reduced levels of glycolytic enzymes hexokinase II and GAPDH, as well as lactate transporting monocarboxylate transporter 1 in ALI, whereas expression of the glucose transporter GLUT1 remained unchanged. Cytochrome *c* oxidase (COX) subunit 5B protein analysis was increased in ALI, although mRNA level remained at constant level. COX activity was assessed using photometric quantification and a three-fold increase was found in ALI. Quantification of glucose and lactate concentrations in cell culture medium revealed significantly reduced glucose levels and decreased lactate production in ALI. In order to evaluate energy metabolism, we measured cellular adenosine triphosphate (ATP) aggregation in homogenised cell suspensions showing similar levels. However, application of the uncoupling agent FCCP reduced ATP levels in ALI but not in SMC. In addition, HIF showed reduced mRNA levels in ALI. Furthermore, HIF-1*α* protein was reduced in the nuclear compartment of ALI when compared to SCM as confirmed by confocal microscopy. These results indicate a metabolic switch in IPEC-J2 cultured under ALI conditions enhancing oxidative phosphorylation and suppressing glycolysis. ALI-induced improvement of oxygen supply reduced nuclear HIF-1*α*, demonstrating a major change in the transcriptional response.

## Introduction

Cell culture models use either the isolation of primary cells or cell lines. Primary cells are non-transformed and not tumour derived. In contrast, the use of continuous growing cell lines allows long-time cultivation using straightforward methods at manageable costs. For clinical and translational research gut epithelial cell culture models should resemble the human *in vivo* situation.

Important morphological features indicating an adequate gut epithelial cell culture model are: (1) development of highly prismatic enterocytes as monolayer, (2) polarised cell growths with a well-defined apical and basolateral cell membrane compartment, (3) microvilli on the apical side, (4) expression of lateral tight junction complexes enabling the epithelial barrier function and (5) desmosomes and zonula adherens between the epithelial cells. A technical prerequisite is a monolayer support (comparable with the epithelial basement membrane) with pores. Within many various continuous cell lines IPEC-1 and IPEC-J2 provide an exceptional option, as they are both originally isolates from new born piglets and are non-transformed, and not tumour derived.^1^ Especially IPEC-J2 cells show morphological and functional similarity to porcine enterocytes. This cell line represents a well-established model for simulation of the human intestinal barrier.^2,3^ IPEC-J2 cells are cubic and partly high prismatic epithelial cells. However, their size and height (proportion of the lateral dimension of a single cell) are dependent on culture conditions such as submerged membrane culture (SMC) or air–liquid interface (ALI) cultures.^4^

Modifying the culture conditions of ALI does not only affect morphological characteristics of the cell lines, but has also impact on their metabolic profile. Kondo *et al.*^5^ as well as Xu *et al.*^6^ demonstrated a significant increase in oxygen turnover in airway epithelial cells of mice and bovine cells applying the ALI protocol.

An important role in the adaptation and coordination of the cellular response to changing oxygen levels is mediated by the hypoxia inducible factor (HIF). HIF is a heterodimeric protein consisting of a *α*- and *β*-subunit, whereas both subunits belong to the helix-loop-helix-PAS family.^7,8^ Dimerisation of both subunits leads to a change in protein conformation and allows its binding to the hypoxia-responsive element within the DNA sequence of target genes.^9–13^ The *β*-subunit is known to be an ubiquitously expressed receptor that is found in excess.^14^ However, HIF-1*α* protein (one isoform of the *α*-subunit) is specific for the HIF-1 protein and shows a complex oxygen-dependent regulation. Thus, HIF-1*α* is instrumental in monitoring the oxygen supply of cell cultures.

Modification of culture conditions resulting in a change of the oxygen available can influence the biochemical and physiological processes in the epithelial cell culture. The aim of the present study was to analyse what effects the ALI culture of IPEC-J2 has on the morphology of the developing cells. A further aim was to demonstrate the impact of an improved oxygen supply as a result of ALI culturing method on the aerobe and anaerobe metabolism in IPEC-J2 cells, and to understand the functional role of HIF-1*α* as mediator of the metabolic adaptation process.

## Results

### Microarray analyses

**Figure 1:**
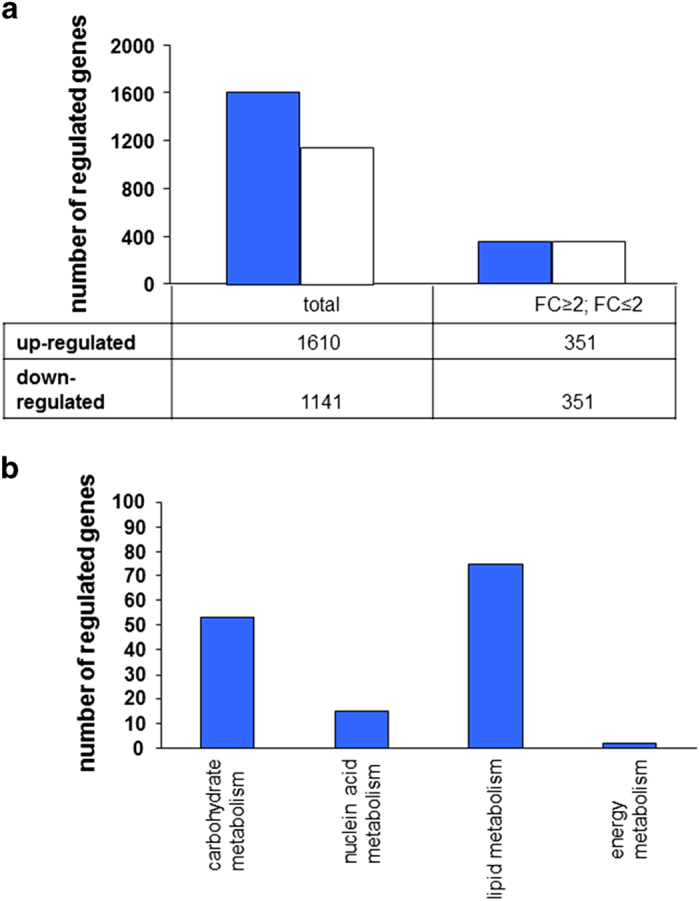
Number of significantly regulated genes (**a**) and identification of significantly regulated pathways of the substrate and energy metabolism (**b**). The microarray analyses showed significant differences in the gene expression profile of IPEC-J2 (**a**). In the ALI cultures a downregulation of 1141 (FC<0) genes and an upregulation of 1610 (FC>0) genes was found. About 351 genes each were up- and downregulated minimum of two-fold (FC⩾2, FC⩽−2), which correlates to a doubling or bisection of the relative expression in the ALI cultures compared to SMC (*P*<0.05). The functional analyses of the microarray identified four different pathways, which are differently regulated in the ALI culture compared to SMC (**b**). The amount of significantly up- and downregulated genes in the pathways is shown. The statistical evaluation was done with a *t*-test (*n*=3; FC⩾2; FC⩽2; *P*<0.5).

A functional clustering of gene transcripts for proteins regulating cellular processes was performed with the DAVID Bioinformatics resources. Functions of the cell cycle, for example, cell proliferation and cell death were affected with 196 and 195 modified transcripts (Figure 1a; FC⩾2, FC⩽−2, *P*<0.05). About 179 transcripts involved in cell development and 94 transcripts assigned to morphology were also found to be altered when compared to gene expression profiles of SMC and ALI. The cell metabolism pathway showed 53 genes in the carbohydrate metabolism, 75 genes of the lipid metabolism and 15 genes of the amino acid metabolism with significant variations in expression (Figure 1b).

### Candidate genes of the microarray analyses

**Figure 2:**
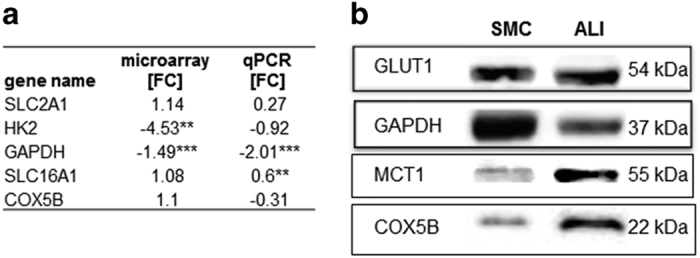
Results of the gene and protein expression. Five candidate genes were examined in our study. Results of microarray analyses and qPCR are shown in (**a**). (**b**) Western blot analyses (*n*=3) showed a lower expression of GAPDH and a higher expression of COX5B and MCT1 in ALI compared to SMC. **P<0.01, ***P<0.001.

There are important genes, which are in part influenced by the ALI culture system (Figure 2). GLUT1 (SLC2A1) encodes for a transmembrane glucose transporter, which plays a key role in regulation of intracellular glucose by modifying specificity and frequency of the transporter in the cell membrane. The gene expression as well as protein expression showed no differences when comparing both culture systems. MCT1 encodes for a monocarboxylate transporter, which facilitates transmembrane transport of lactate and pyruvate, and thereby regulates the intracellular milieu. In western blot analyses, we detected a higher MCT1 protein expression in the ALI cultures than in SMC. In contrast, qPCR analyses resulted in a downregulation of MCT1 in the ALI cultures. This goes along with the data of HK2 and GAPDH. GAPDH was also reduced in the western blot analyses of ALI cultures. Cytochrome *c* oxidase5B (COX5B) protein was higher in the ALI cultures (the gene expression was unaltered).

### ALI cultures improve the oxygen supply and increase COX activity

**Figure 3:**
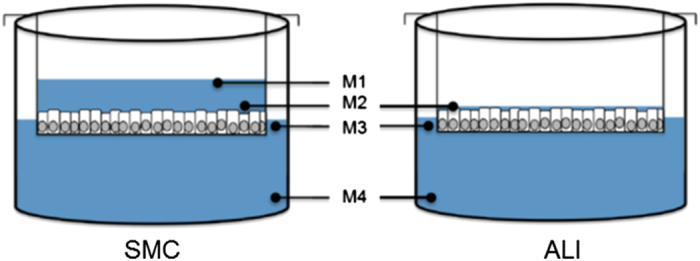
Measure points for analysis of oxygen supply.

**Figure 4:**
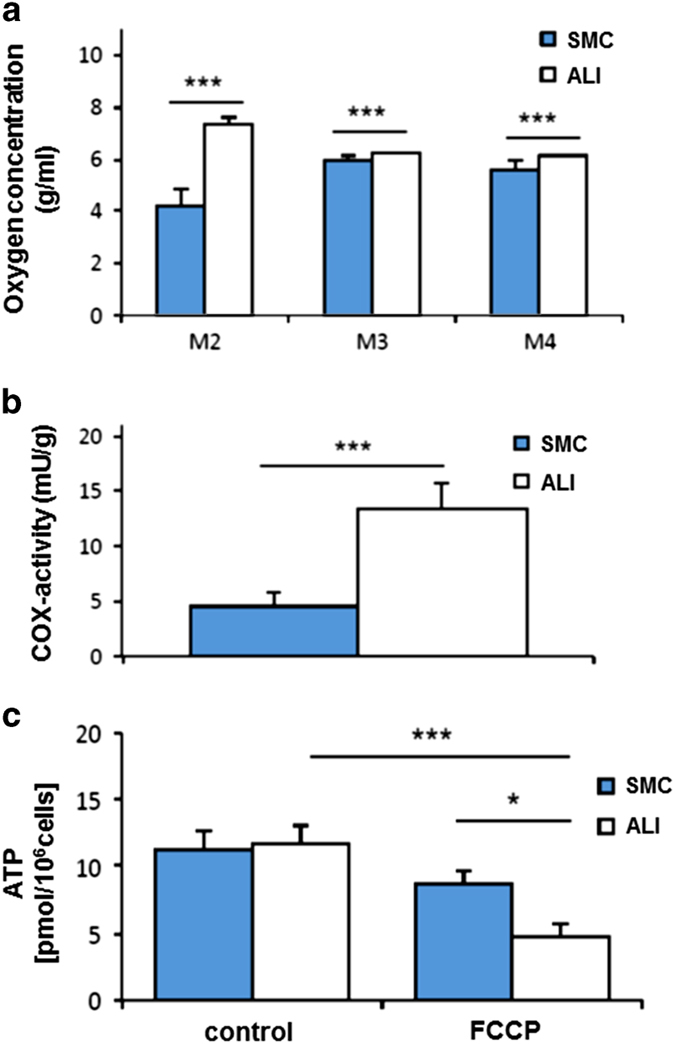
Oxygen supply, COX activity and ATP content. All measure points showed a significant higher oxygen concentration in ALI culture than in SMC (**a**). In comparison to SMC, ALI cultures showed a significant higher cytochrome *c* oxidase (COX) activity ((**b**), ***P<0.001). No differences were found in the ATP content with the focus on SMC and ALI cultures (**c**). The treatment of both cultures with FCCP (10 *μ*M; 24 h) resulted in a significant decrease of the ATP content in ALI cultures but not in SMC (***P<0.001).

The oxygen consumption was measured at three different points within the transwell system (Figure 3). At all survey points, a significantly higher oxygen concentration was found in ALI cultures than in SMC (Figure 4a). Furthermore, COX, the complex IV of the mitochondrial respiratory chain (catalysing the reduction of oxygen to water) showed a higher enzyme activity. The same was true for the cytochrome *c* activity in the ALI cultures; here an increase of 200% compared to SMC was observed (Figure 4b; *P*<0.001).

### Increased application of the respiratory chain in ALI cultures

Comparing SMC and ALI cultures no differences in the adenosine triphosphate (ATP) content were observed (Figure 4c). After application of FCCP (decoupler of the respiratory chain) to the transwell system, a significant decrease in the ATP content in the ALI cultures (*P*<0.001) was detected, while the ATP content in SMC cultures remained at stable level.

### Higher glucose consumption and lactate production in SMC

**Figure 5:**
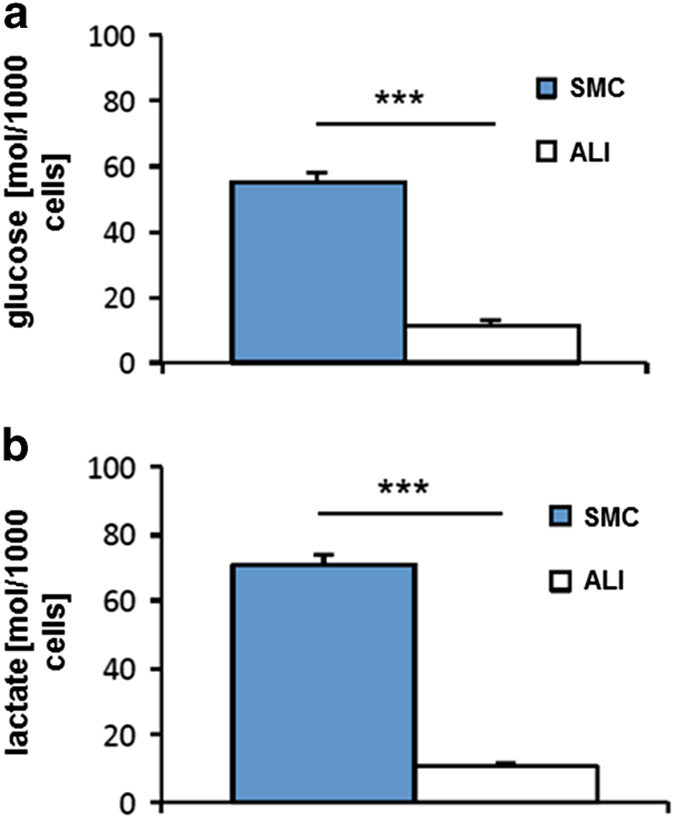
Glucose consumption and lactate production. IPEC-J2 cells in SMC showed a significant higher glucose consumption (**a**) than ALI cultures (***P<0.001). The same results were found with the focus on lactate production (**b**) (***P<0.001).

Figure 5a shows the glucose consumption of IPEC-J2 in SMC and ALI cultures. In our study, we found a significant higher glucose consumption of SMC in comparison to ALI cultures (*P*<0.001). This results in ALI cultures over 5 days in only 21% of the glucose consumption measured in SMC. Furthermore, the lactate production shows according results (Figure 5b). ALI IPEC-J2 cultures produced only a sixth part of the amount of lactate produced by SMC-cultured IPEC-J2 (*P*<0.001).

### Conventional culture induces hypoxia

**Figure 6:**
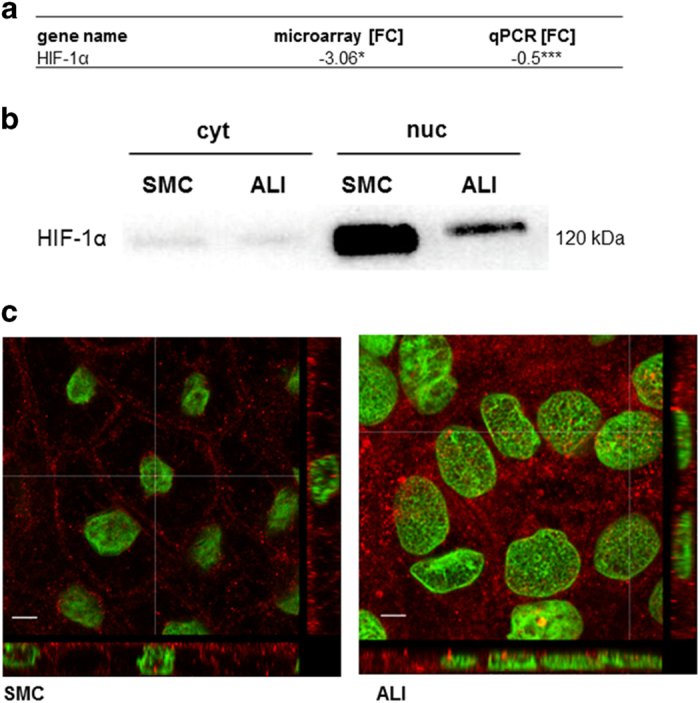
Gene and protein expression of HIF-1*α*. HIF-1*α* showed a significant decrease in the qPCR (*n*=5) and microarray analyses (*n*=3) (**a**). In the next step, nuclear and cytoplasmatic proteins were separated. A higher content of HIF-1*α* was detected in the nuclear fraction of SMC in comparison to ALI cultures (**b**). This was confirmed by confocal microscopy (**c**; Dapi (green) and HIF-1*α* (red)).

In the microarray analyses as well as in qPCR, we found a downregulation of HIF-1*α* in the ALI culture compared to SMC (Figure 6a). This was confirmed by western blot analyses. When comparing nuclear protein density of HIF-1*α*, we found a remarkably higher content in SMC than in ALI cultures. Furthermore, a higher content of HIF-1*α* was detected in the nuclear compartment than in the cytoplasmatic compartment in ALI culture (Figure 6b), – a result confirmed by confocal microscopy (Figure 6c).

### Downregulation of HIF-1*α*-induced genes in ALI cultures

HIF-1*α* is known to activate multiple genes involved in glycolysis under hypoxic conditions including GAPDH, HK2, ALDOC and ENO1. All these genes were significantly downregulated in the ALI cultures compared to SMC (*P*<0.001; Table 1). However, SLC2A1, HK1, SLC16A4 and PFKL, which are also known to be HIF-induced genes, showed no differences.

## Discussion

Hypoxia in tissues arises as a consequence of an inadequate oxygen supply and goes along with the disturbance of biological function.^15^ An acute hypoxic status occurs based on the fluctuation of the oxygen transport caused by variability in the tissue perfusion.^16^ A chronic hypoxic status is established by permanent critical shortage, which may be caused by a tumour^17^ and is called diffusion-limited hypoxia.^18^ The gastrointestinal tract accounts for 20–25% of human body oxygen turnover due to the energy-consuming transport processes along the epithelial barrier.^19^ The variability of the oxygen turnover arises from oxygen demand of the food. In a pig-feeding study, an increase of the intestinal oxygen uptake up to 33% was detected 60 min postprandial compared to basal oxygen uptake. After a meal, a higher portion of oxygen is extracted from the blood to cover the oxygen demand of the intestinal cells.^19^ In addition, feeding evokes an increase of the intestinal blood stream up to 200% compared to the basal flow rate.^20^ In our study, we altered the long-term oxygen supply of the IPEC-J2 cells by applying the ALI culture protocol. In SMC cultures, oxygen availability to IPEC-J2 is limited by the diffusion speed of oxygen through cell culture medium. Reducing the apical diffusion layer – that is the cell medium volume on the apical side – increases the oxygen content at the medium–cell interface.^21^ Higher oxygen levels stimulate its turnover.^22^ The use of cell culture transwell insert wells enables IPEC-J2 cells to extract oxygen bidirectional, whereas the change in the apical oxygen using the ALI culture results in a marked increase of oxygen quantity also in the basal medium layer. This was confirmed in a significant lower oxygen concentration in the basal measure points of SMC culture compared to ALI culture. In comparison, a reduction of hypoxia was also shown by Heinis *et al.*,^23^ who cultivated primary cells of rats on collagen as SMC culture or ALI culture. In their experiment hypoxic regions of the culture were identified using pimonidazol and anti-Mab1-antibody.^24^ After 24 h incubation, hypoxic regions were found in SMC collagen cultures but not in ALI cultures. The role of oxygen deficiency is also discussed with respect to the pathogenesis of Morbus Crohn and Colitis ucerosa: obviously hypoxia increased the permeability of the intestinal barrier, resulting in a putative pathomechanism of the mucosal inflammation.^25,26^

The biochemical and morphological evidence of an increase of nuclear HIF-1*α* – detected in our SMC cultures of IPEC-J2 cells – is an important indication of the exposition of the cells to oxidative stress, comparable to the oxygen deficit as it occurs in tumours.^27^ Furthermore, the morphological detection of HIF-1*α* in the histology of the cell cultures may serve as prognostic factor in the clinical progress of different tumours such as lung, breast and bladder cancer. Nevertheless, it is necessary to discuss whether a decreased activity of the transcription factor HIF-1*α* acting in the ALI cultures is responsible for the modulation of the cell metabolism of IPEC-J2 cells.

Therefore, nine representative HIF-1*α*-target genes involved in glucose and lactate transport were evaluated with the focus on their expression profile in ALI cultures. Five of them showed a reduced expression in ALI culture pointing to a HIF-dependent regulation. Moreover, the reduction of glycolytic enzymes and the downregulation of HIF-1*α* demonstrates the switch in the energy metabolism of IPEC-J2 in ALI cultures towards oxidative phosphorylation, based on the large amount of oxygen available under this culture condition. On the other hand, hypoxia results in glycolysis and lactate production. To maintain the balanced energy metabolism under hypoxia, an inverse correlation is necessary, that is, an increase of the inefficient energy supply by glycolysis under decreased oxygen availability,^28,29^ finally leading to elevated lactate production. Hence, quantification of glucose turnover and lactate production with respect to the ATP content allows major conclusions about the anaerobic or aerobic metabolism in cell culture systems. Kondo *et al.*^5^ demonstrated a negative correlation between lactate production and the apical medium layer thickness in the cell culture. . The comparison of SMC cultures and ALI cultures in our experiments showed a remarkable reduction of lactate production in ALI cultures. This is clear evidence for an increased activity of oxidative phosphorylation, in comparison to earlier studies.^5,30^ In our ALI cultures of IPEC-J2 cells we detected also a reduced glucose turnover and a reduced lactate production. Furthermore, we could not find any differences in the ATP content between both culture methods, but the use of the decoupler of the respiratory chain – FCCP – showed a significant decrease of ATP in the ALI culture but not in SMC cultures. In addition, a higher COX activity was found in ALI cultures compared to SMC cultures. The synopsis of all these results points to a preferred use of the aerobic metabolism in the IPEC-J2 ALI cultures.

## Conclusion

In conclusion, based on the ALI cultivation an optimised oxygen supply of IPEC-J2 cells is evident. The induction of an aerobic ATP generation associated with the reduction of the glycolytic capacity is demonstrated, with HIF-1*α* being the important mediator. Our observations are of major importance for all cell culture systems, especially for intestinal epithelial cell cultures. It is essential to develop epithelial cell biology in the light of the two metabolic pathways of ATP production in the cells – namely the glycolysis and the oxidative phosphorylation, which is in terms of energy much more efficient.

## Materials and Methods

### Cell culture protocol

Intestinal epithelial cell line IPEC-J2 (DSMZ-Nr. ACC 701) was cultured as described by Nossol *et al.*^4^ Cells were seeded with a density of 1×10^5^ cells/ml on the upper side of transwell system. Size of transwell system was selected according to experimental design (pore size 1 *μ*m, diameter 10 mm/15 mm, ThinCerts, Greiner Bio One, KremsmÃ¼nster, Germany) and inserts were placed in corresponding dishes. Cell culture medium was based on DMEM/HAMs F12 and supplemented with 5% FCS, 16 mM HEPES, 5 ng/ml EGF and Vol. 1% ITS (PAN, Aidenbach, Germany). IPEC-J2 cells were grown at 39 °C in an incubation atmosphere of 95% relative humidity and 5% CO_2_. For SMC cell culture medium was applied to apical and basolateral compartment. After 10 days of cultivation, FCS supplement in culture medium was reduced stepwise every second day (2.5, 1, 0%) and cells were cultured for 21 more days in FCS-free cell culture medium. For ALI culture IPEC-J2 were cultured analogue to SMC for 10 days with subsequent FCS reduction, though when applying FCS-free medium, apical cell culture medium was withdrawn (day 14) and cells were cultured for 21 more days with no more than a thin liquid film on apical side. Cell culture medium was changed every 2 days. For quantification of glucose and lactate cell culture medium was left on cells for 5 days before retrieving samples.

### Transepithelial electric resistance

Confluence was assessed by visual inspection of membrane integrity and quantification of transepithelial electric resistance (TEER) using MILLICELL-ERS (Millipore, Darmstadt, Germany). For TEER measurement pre-warmed medium was added to apical compartment of ALI for the duration of measurement only (maximum duration 5 min). In between the measurements the electrode was disinfected using 70% ethanol, washed in ampuwa and calibrated in pre-warmed FCS-free cell culture medium. TEER was assessed once a week. As a criterion of inclusion for further experiments TEER must not fall below 0.5 kΩcm^2^ at any time during cultivation.

### RNA isolation

For microarray analysis and quantitative real time PCR IPEC-J2 were cultured on ThinCerts with 10 mm diameter. After withdrawal of apical and basolateral medium, cells were covered with TRIzol reagent (Invitrogen, Waltham, MA, USA) as described by the manufacturers protocol and scraped of the membrane. After adding chloroform to the cell lysate, supernatant was extracted and RNA was precipitated using isopropanol alcohol. Using 75% ethanol the RNA was purified and stored in RNA-free water peqGOLD (Peqlab, Erlangen, Germany) at −80 °C until further processing.

### Microarray analysis

For microarray analysis the isolated RNA of at least three independent experiments was further purified employing RNeasy Kit (Qiagen, Hilden, Germany) and quantity was determined using NanoDrop ND-1000 spectrophotometer (Peqlab).

The array analysis was performed as described by Diesing *et al.*^31^ before. Briefly, Gene Chip Porcine Genome Array (Affymetrix, Santa Clara, CA, USA) was loaded with 500 ng of purified RNA and analysed using Affymetrix GCOS 1.3 software (Waltham, MA, USA). At first, data were inspected for quality before further preliminary processing, that is, background correction and normalisation were performed. Differentially expressed genes were identified and statistically evaluated using R statistical language (Bioconductor Packages; https://www.bioconductor.org) and statistical significance was examined by applying *t*-test (*P*<0.05). For functional analysis significantly up- and downregulated genes were assigned to cellular pathways using DAVID bioinformatics resources.

### Quantitative real time PCR

For quantitative real time PCR at least five independent experiments were carried out. At first, RNA content was measured using Qubit RNA Assay Kit (Life technologies, Waltham, MA, USA). Quantitative real time PCR was performed using SensiFast TM SYBR/No-ROX One Step Kit (2 ng RNA; 10 pmol of each primer (Supplementary Table A); Bioline, Bochum, Germany) according to manufacturer’s protocol. *β*-Actin served as housekeeping gene. RNA reverse transcription, amplification and quantification was operated using qTower (Analytik Jena AG, Jena, Germany). For reverse transcription samples were heated up to 45 °C for 10 min followed by the activation of polymerase (95 °C, 2 min) and 40 cycles of denaturation (95 °C, 5 s), and annealing with elongation (varying temperature according to the primer, 20 s) in alternating order. Experiments were carried out in sets of three replicates, whose results were averaged and further mathematically processed using ΔΔ-CT method.

### Western blot

For protein analysis IPEC-J2 were cultured in ThinCerts of 15 mm diameter. At least three independent experiments were performed. Medium was withdrawn, cells were washed in PBS and SDS loading buffer was added (1 M Tris base pH 6.8, Vol. 10% glycerol, Vol. 2% SDS, Vol. 0.005% bromophenol blue, Vol. 5% *β*-mercaptoethanol). For protein denaturation the lysate was heated up to 95 °C for 5 min. Quantification of protein content was performed using Molecular probes Qubit Protein Assay Kit and Qubit 2.0 Fluorometer (both Invitrogen) in accordance to the manufacturers’ protocol.

For western blot 40 *μ*g of protein sample as well as Page Ruler prestained protein ladder (SM0671; Fermentas, Waltham, MA, USA) were placed in parallel order on SDS polyacrylamide gel. After electrophoresis, samples were transferred to 0.45 *μ*m PVDF membrane by semidry electro blotting using Trans-Blot SD Semi Dry Transfer Cell (Bio-Rad, Munich, Germany). Protein detection was performed employing BM Chemiluminescence Western Blotting Kit (Mouse/Rabbit) by following the manufacturer’s instructions. Primary antibodies were utilised to identify specific proteins: rabbit anti-GLUT1 (1 : 500, Bioss, Woburn, MA, USA), mouse anti-GAPDH 1 : 2000 (Cell Signaling, Cambridge, UK), rabbit anti-MCT1 1 : 1000 (Bioss), rabbit anti-COX5B 1 : 1000 (Abgent, Danvers, MA, USA), mouse anti-HIF-1*α* 1 : 500 (Novus Biologicals, Abingdon, UK), mouse anti-*β*-actin 1 : 40 000 (Sigma-Aldrich, Munich, Germany). Blots were visualised by MultiImage Light Cabinet (Alpha Innotech, Kasendorf, Germany).

In order to detect protein content in distinct intracellular compartments, protein extraction was performed using NE-PER Nuclear and Cytoplasmic Extraction Reagents (Thermo Scientific, Waltham, MA, USA). Following the manufacturer’s instructions, IPEC-J2 were liberated from cell culture medium and incubated in Tyrpsin/EDTA for 20 min. Afterwards cells were re-suspended in 500 *μ*l of FCS-free cell culture medium and scraped of the membrane. Suspensions of three inserts were pooled and centrifuged (500×*g*, 5 min). After discarding the supernatant, the remaining pellet was re-suspended in 1 ml PBS/protease inhibitor (Roche, Basel, Switzerland). Cell count was performed and suspension was centrifuged (500×*g*, 3 min, 4 °C) once more before discarding the supernatant. CER I (−20 °C, volume in accordance to cell count) was added to the lysate and vortexed (15 s). After an incubation period of 15 min on ice, CER II (−20 °C, volume in accordance to cell count) was added resulting in the liberation and solution of cytoplasmic proteins in the supernatant while maintaining nuclear integrity. Supernatant was withdrawn and stored at −80 °C until further processing. The remaining pellet was re-suspended using NER I solution leading to the liberation of nuclear protein fraction in the supernatant, which was stored in analogous manner. For further processing, protein content in samples was assessed and 40 *μ*g of extracted protein was admixed with equimolar amount of SDS loading buffer before electrophoretic separation occupying an 8% SDS polyacrylamide gel.

### ATP quantification

For ATP analysis IPEC-J2 were seeded on ThinCerts of 10 mm diameter. Medium was withdrawn and membranes were liberated from framework. Cells were covered with boiling 4 mM EDTA/100 mM Tris buffer and scraped of the membrane before the lysate was incubated at 100 °C for 2 min. After centrifugation (1000×*g*, 60 s) 50 *μ*l of supernatant were transferred as triplets onto a white 96-microplates (Greiner Bio One) and plates were kept on ice until further processing. ATP quantification was carried out using ATP Bioluminescence Assay Kit CLS II (Roche) according to manufacturer’s protocol occupying Tecan M200 (Tecan, MÃ¤nnedorf, Switzerland). For FCCP treatment, 10 mM 2-[[4-(trifluoromethoxy)phenyl]hydrazinylidene]-propanedinitrile was subjoined to basolateral compartment and incubated for 24 h prior to ATP investigation.

### Quantification of glucose and lactate

In order to measure the oxygen content within the cell culture medium IPEC-J2 were grown on ThinCerts of 15 mm diameter, but medium was unaltered within the final 5 days of cultivation. After termination of cell culture duration, cell culture medium was fully withdrawn from the cells and transferred into separate tubes according to compartment, distinguishing upper (apical) and lower (basolateral) compartment. Pre-warmed cell culture medium (FCS-free) was added to remaining cells, cells were scraped of the membrane mechanically and lysate was stored separately. All samples were stored on ice until measurement. Cell-free cell culture medium was used as blank. Glucose and lactate concentrations were determined immediately using Cobas C 501 (Roche) as well as reagents of test systems GLUC2 and LACT2 (Roche), respectively. The differences in glucose and lactate concentration between blank and samples were considered as glucose consumption and lactate production, respectively.

### Oxygen analysis

In order to measure the oxygen content within the cell culture medium IPEC-J2 were grown on ThinCerts of 15 mm diameter but medium was unaltered within the final 5 days of cultivation. For quantification, oxygen sensor Microx TX3 (PreSense, Regensburg, Germany) was calibrated prior to measurement according to manufacturer’s instructions. Afterwards, both sensors detecting oxygen and temperature were placed at four and three different checkpoints in SMC and ALI culture, respectively: SMC: M1 apical compartment at gas–medium interface, M2 apical compartment at medium–cell interface, M3 basal compartment at gas–medium interface, M4 basal compartment right upon the dish. In ALI culture only M2-M4 was examined (Figure 3). Measurements (10 s, interval 250 ms) were repeated three times for at least five independent experiments.

### Cytochrome *c* activity

IPEC-J2 cells were seeded on ThinCerts of 15 mm diameter and cell culture medium was withdrawn at the end of cultivation. Membranes were liberated from framework and cells were covered with 250 mM sucrose, 1 mM EDTA, 0.1% BSA in aqua dest. before being scraped of the membranes. The lysate was transferred into tubes and homogenised using Potterhomogenisator Tissue Grind tube size 20 (Kimble Chase, Gerresheimer, Vineland, NY, USA) while stored on ice. After centrifugation (631×*g*, 4 °C, 5 min) supernatant was extracted and centrifuged once more (5100×*g*, 4 °C, 4 min). The retrieved pellet was re-suspended in 500 *μ*l of 250 mM sucrose solution and centrifuged (12 400×*g*, 4 °C, 10 min). After discarding, the supernatant, the pellet was re-suspended once more in 250 mM sucrose solution, centrifuged (12 400×*g*, 4 °C, 2 min) and liberated from supernatant. Afterwards the remaining pellet was resolved in 10 mM Tris/HCl with supplement of 250 mM sucrose and stored on ice until further processing. Prior to examination the photometer Smart SpecTM300 (Bio-Rad) was calibrated using 10 mM Tris/HCl supplemented with 120 mM KCl. For photometric measurement, 10 *μ*l of sample were mixed up with 5 *μ*l of cytochrome *c* DTT solution (2.7 mg cytochrome *c* from equine heart, 5 *μ*l 0.1 M Dithiothreitol (both Sigma, St Louis, MO, USA) in 1 ml Aqua dest.) and 95 *μ*l of 10 mM Tris/HCl with 120 mM KCl. Absorption was measured at 550 nm for the duration of 60 s at an interval of 10 s. In addition, the absorption of a blank (10 *μ*l 10 mM Tris/HCl with 250 mM sucrose, 3.5 *μ*l cytochrome *c* DTT solution, 95 *μ*l 10 mM Tris/HCl with 120 mM KCl) was assessed. Enzyme activity was calculated as shown by Equation (1):

$$Cytochromecoxidaseactivity(U/µl)=\frac{(meanabsorption\left(sample\right)-meanabsoprtion\left(blank\right))\times 0,105}{10µl\times 21,84}$$

### Confocal microscopy

Multichannel stacks of immunocytochemical-stained tissue sections were sequentially recorded using a Leica SP5 confocal microscope (Leica Microsystems, Wetzlar, Germany), equipped with argon, DPSS, HeNe and titanium:sapphire lasers and sensitive hybrid photomultipliers (HYD). Regions of interest were scanned by using a ×63 oil immersion objective with a numerical aperture of 1.4 (HCX PL APO CS 63.0×1.40 OIL UV). The diameter of the confocal pinhole was set to 95.5 *μ*m (=Airy 1) and images were taken at 1024×1024 pixel resolution with zoom factor 4 and a scan rate of 700 Hz with line average of 4. Usually, 43 optical planes were scanned in axial direction with a step size between two focal planes of 0.17 or 0.25 *μ*m. These settings resulted in an image volume of 61.5 *μ*m (x)×61.5 *μ*m (y)×7 *μ*m (z) or 10.6 *μ*m (z) with a voxel size of 60.2 nm (x)×60.2 nm (y)×168 nm (z) or 252 nm (z) at 8-bit gray scale resolution.

For spectral filtering the laser microscope was equipped with acousto optic modulators, called acousto optical tunable filter (AOTF). An AOTF consists of an optically transparent crystal with which intensity and wavelength of incident light can be adjusted continuously. Furthermore, the system contained an AOBS (acousto optical beam splitter) that separates the detection beam path from excitation. The fluorophores of the secondary antibodies were sequentially excited with a wavelength of 561 nm (DPSS laser) or 594 nm (HeNe 594 laser) for image acquisition of Alexa 594 in the spectral range of 606–700 nm (channel 1). For excitation of the nuclear staining a titanium:sapphire laser tuned to 780 nm was used in two-photon-excited mode and emission was detected in the spectral range of 433–520 nm (channel 2). With this sequential excitation setting any crosstalk between channels could be excluded.

To address the question of a nuclear localisation individual focal planes or image stacks were checked in xz or yz direction for in the allocation of immune markers.

### Statistical analysis

Statistical analysis was carried out using SPSS 15.0. (Ehningen, Germany) Results were tested for normal distribution using Kolmogorov–Smirnov test or Shapiro–Wilk test in accordance to sample size. Statistical significance was assessed using *t*-test unless otherwise stated (*P⩽0.05, **P⩽0.01, ***P⩽0.001).

## Figures and Tables

**Table 1:** Analyses of HIF-1*α*-induced genes

*Gene name*	*Microarray (FC)*
SLC2A1	1.14
HK1	1.5
HK2	−4.53**
PFKL	−1.22
GAPDH	−1.49***
PGK1	−1.39*
ENO1	−1.75**
SLC16A1	1.34

*P<0.05, **P<0.01, ***P<0.001.
