# Supplementary material for: Air–liquid interface enhances oxidative phosphorylation in intestinal epithelial cell line IPEC-J2
Source: Cell Death Discov. 2017 Feb 27;3:17001–. doi: 10.1038/cddiscovery.2017.1 (PMC5327501; doi:10.1038/cddiscovery.2017.1)
Supplement: Supplemental Information [file cddiscovery20171-s1.doc]

| **name** | **function** | **sequence 5’-3’ primer**  **left** | **sequence 3’-5’primer**  **right** | **temperature [°C]** | **efficiency**  **[%]** |
| --- | --- | --- | --- | --- | --- |
| Actin | β-Actin | tgcactttattgaactggtctca | gtatgaagttcaacgccctgt | 61,0 | 115 |
| COX5B | Cytochrom-c oxidase subunit 5B | tgatgaggagcaggcga | gtcggagtccatggttcctt | 56,5 | 109 |
| GAPDH | Glycerinaldehyde-3-phosphate-dehydrogenase | acccagaagactgtggatgg | ttccagtagggactcgagtt | 56,5 | 128 |
| GLUT1 | Glucosetransporter 1 | gagccctgcctagacacttg | ccacctcttggggtagaaga | 60,0 | 89 |
| HIF1a | Hypoxia-inducible factor | cagctatttgcgtgtgagga | aaaccatccaaggctttcaa | 60,0 | 94 |
| HK2 | Hexokinase 2 | ttgaacagcagacaccgtcta | gcattctccagcgttctttc | 60,0 | 108 |
| MCT1 | Monocarboxylate-transporter 1 | tccatcatgttggctgtcat | gaaggaagctgcaatcaagc | 60,0 | 86 |

**Table A**
